# Report on the Relations of the Veterinary Profession to the Department of Police1Read at the banquet before the Chicago Veterinary Society, Chicago, June 10, 1897.

**Published:** 1897-07

**Authors:** James Robertson


### REPORT ON THE RELATIONS OF THE VETERINARY PROFESSION TO THE DEPARTMENT OF POLICE OF THE CITY OF CHICAGO.[1]

Chapter 51, Article I., paragraph 1477 of the Municipal Code provides for the appointment of veterinary surgeons, but makes no mention of their rank or dutes. The late Chief's report, December 31, 1896, provides for one veterinary surgeon and one assistant. So also does ex-Comptroller Wetherill's estimate for 1897: Said appointees to receive for the first position $1800, and for the second $1000 per annum. There were two assistants, but where the second was placed the report does not say. For the past two years the positions were filled by qualified men, graduates of recognized colleges. The present are unqualified.

There are about 235 horses in the department, 4 for each wagon or ambulance, 4 for telegraph wagon, 1 each for Mayor, Chief, Assistant Chief, Captains, and veterinarians.

There is a veterinary hospital for the care of sick and disabled animals, belonging to the department. The work of the police department is very hard on horses. They have to attend every fire, and this means only about 1 per cent. of their work.

The appointment of veterinarians for the department changes with the administration. Sometimes the Mayor names the candidates, and sometimes the Chief of Police. Although the code provides for the appointment of veterinary surgeons, there have been no veterinarians appointed, the "powers that be" evidently believing in the maxim "that a little knowledge is a dangerous thing in connection with the department of police." As far as we can learn, this is the only instance where the profession is entirely ignored in the care of horses belonging to large cities. However, the responsibility for this condition of affairs, we think, rests largely with ourselves. We have allowed a commercial aspect to govern our conduct;

[1] Read at the banquet before the Chicago Veterinary Society, Chicago, June 10, 1897.

in fact, proclaimed to the public that we were conducting our affairs on business principles, and the public have accepted our proposition, and met us on the same terms, and if, in the competitive struggle, we have been crowded out we have no cause to complain.

The organization of this Association is the first step toward raising the profession above the level of a business. Surely, our training and relation to the public weal would entitle us to a higher plane than this, but we have to clear our pathway ourselves, and our present experience should be a valuable lesson and stimulus toward organization and work. We should not only jealously guard each other's professional reputation, but do our duty as citizens in municipal affairs, and in this way wield a united influence that cannot be ignored. Respectfully submitted,

James Robertson.

